# The complete mitochondrial genome of the *Kaloula verrucosa* (Anura: Microhylidae) and phylogenetic analyses

**DOI:** 10.1080/23802359.2018.1467238

**Published:** 2018-04-28

**Authors:** Shouhong Wang, Lusha Liu, Meihua Zhang, Jianping Jiang

**Affiliations:** a Chengdu Institute of Biology, Chinese Academy of Sciences, Chengdu, China;; b Department of Herpetology, Chengdu Institute of Biology, Chinese Academy of Sciences, Beijing, China

**Keywords:** Microhylidae, *Kaloula verrucosa*, mitogenome, China

## Abstract

The first complete mitochondrial genome of a *Kaloula verrucosa* frog was characterized in this work. The mitogenome was 17,061 base pairs (bp) in length, containing 13 protein-coding genes (PCGs), 2 rRNA genes, 22 tRNA genes, and a control region (D-loop). The overall base composition was 29.65% A, 30.77% T, 25.41% C and 14.17% G. Besides, the gene arrangement was identical to that observed in vertebrates. Five of 13 PCGs (*COII*, *ATP6*, *COIII*, *ND3* and *ND4*) were ended with incomplete stop codon T. Except for *ND6* gene encoded on L-strand, all other PCGs were encoded on H-strand. The non-coding region was 1665 bp in size, which was heavily biased to A + T (65.77%). Additionally, we found mitogenome size of all sequenced *Kaloula* species were bigger than that of *Microhyla* species, which was ascribe to the difference of D-loop size. Phylogenetic analysis showed that *K. verrucosa* was the sister species of *Kaloula regifera*. This work will provide basic molecular data for further molecular evolution and phylogenetic research of *K. verrucosa* and other microhylids.

*Kaloula Gray* 1831 is a genus of 17 species, showing a broad distributional range in Korea and northern China to Lesser Sundas and Philippines, Bangladesh, and India, controversially in Nepal (Frost 2018). However, only three species (*Kaloula pulchra* AY458595, *Kaloula borealis* NC_020044, *K. rugifera* KP682314) of this genus were determined the complete mtDNA sequence (Hwang and Lee 2012; Wu et al. 2014; Deng et al. 2016). Therefore, more complete mitochondrial genome species would improve our understanding for further genetic research and conservation of this genus. *Kaloula verrucosa* Boulenger, 1904 is an endemic species of China, occurring in Sichuan, Yunnan, and Guizhou (Yang and Wu 2004; Jiang et al. 2016; Jiang et al. 2016). Here, Genomic DNA extraction, mtDNA amplification and sequencing were from leg muscle preserved in 95% ethanol of a single *K. verrucosa* specimen collected from Huili, Sichuan (26.74198°N and 102.48511°E) and the specimen was stored in department of Herpetology of Chengdu Institute Biology (number 20090351). The amplified primers were used as described by Zhang et al. (2013) and amplified methods were performed according to Khatiwada et al. 2017.

The total length of complete mitogenome was 17,061 bp (GenBank accession no. MG962359), which included 13 PCGs, two ribosomal RNA genes, 22 transfer RNA (tRNA) genes, and a control region (D-loop). The gene organization was similar to that observed in most microhylids (Wu et al. 2014; Deng et al. 2016; Wang et al. 2016). The overall base composition was 29.65% A, 30.77% T, 25.41% C, and 14.17% G. The AT content (60.42%) was much higher than that of GC (39.58%). *ATP8* gene (165 bp) was the shortest while *ND5* was the longest (1806 bp) among the 13 PCGs. Except for *ND6* and 8 tRNA genes (*tRNA-Pro*, *tRNA-Gln*, *tRNA-Ala*, *tRNA-Asn*, *tRNA-Cys*, *tRNA-Tyr*, *tRNA-Ser*, and *tRNA-Glu*) encoded on L-strand, the remaining 28 genes were encoded on H-strand. Most of the PCGs were terminated with a complete stop codon (TAG, AGG, TAA or AGA), however, five genes (*COII*, *ATP6*, *COIII*, *ND3* and *ND4*) were stopped with incomplete T. Each typical tRNA cloverleaf secondary structure is predicted by tRNA-Scan Web Server (Lowe and Chan 2016). Total 22 tRNA genes, with the size ranging from 57 bp to 73 bp, were interspersed along the whole genome, and most of the tRNAs could form a cloverleaf structure. The putative origin of L strand replication (O_L_), with size of 29 bp between the *tRNA-Asn* and *tRNA-Cys* genes, could be folded into a stem loop of secondary structure, which was similar to that of other vertebrates (Deng et al. 2016). The D-loop region (1665 bp), located between *Cytb* and *tRNA-Leu*, was heavily biased to A + T (65.77%). In addition, the mitogenome sizes of sequenced *Microhyla* species (Mean ± SD, 16,729.29 ± 19.49 bp) were smaller than that of *Kaloula* species (17,031.5 ± 151.09 bp), which might be ascribe to the difference of D-loop size (*Microhyla*: 1341.29 ± 18.06; *Kaloula*: 1629.75 ± 152.48). D-loop region which was the most variable part may play a key role in molecular evolution and phylogenetic research.

Neighbour-joining algorithm tree was constructed based on 11 complete mtDNA sequences of available Microhylidae (Figure 1). The result showed that *Microhyla*, and *Kaloula* were from two strong monophyletic clades and *K. verrucosa* is the sister species of *K. regifera*.

**Figure 1.**
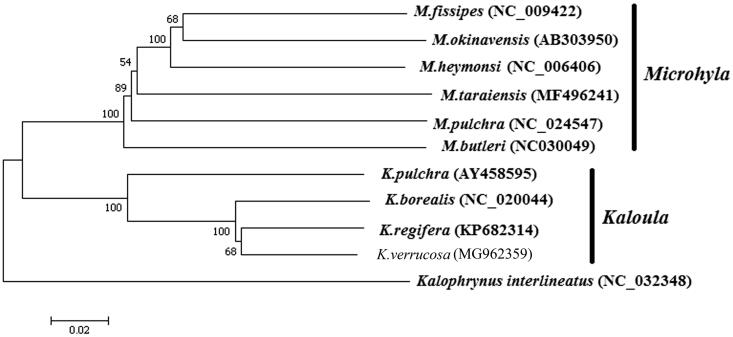
Neighbour-joining algorithm tree was constructed based on 11 complete mitochondrial genome sequences. The branches were validated by bootstrap analysis from 1000 replications and the numbers in branch nodes were bootstrap support values. The position of *K. verrucosa* was not shown in bold and *Kalophrynus interlineatus* was used as outgroup.
